# Proceedings of the Chicago Medical Society

**Published:** 1868-03-15

**Authors:** 


### PROCEEDINGS OF THE CHICAGO MEDICAL SOCIETY.

*Abscess of the Brain—Disease of Kidneys.*—Dr. Fenn exhibited the kidneys of a male patient, twenty years of age, who had been brought to the Cook County Hospital in a moribund condition. No satisfactory history of the case could be obtained. The urine was highly albuminous, the cerebral symptoms usually attending uræmic poisoning, being present.

The autopsy revealed great congestion of the kidneys, especially of the pyramids.

The brain was extensively covered with thick layers of lymph. On removing the cerebrum, a distinct fluctuation was observed in the right middle lobe. An incision disclosed an abscess, containing about an ounce of dark, fœtid, purulent fluid.

*Internal doses of Chloroform in Delirium Tremens.*—Dr. McDonald reported a case of delirium tremens, in which the patient had not slept for three days and nights. The ordinary remedies had failed to give relief, possibly in consequence of constant vomiting, which had prevented the patient from retaining them. There was great nervous prostration.

A drachm of *chloroform*, followed every three hours with a much smaller portion, soon restored quiet and rest. The disease speedily subsided, leaving, however, a very painful irido choroiditis of the left eye, which was subsequently relieved by an iridectomy.

*Olive Oil in cases of Gall Stones.*—Dr. Hatch exhibited several large gall stones, which had been dejected after large doses of olive oil. The patient had lost a mother and elder sister several years before from hepatic disease, in which the passage of gall stones was one of the principal symptoms.

The patient, a female about fifty years of age, had suffered from frequent attacks of illness, with symptoms of gall stones. In June last, she suffered severe symptoms of the disease, which were relieved, after taking each night for three nights, a third of a large common bottle of olive oil. The several very large stones presented to the society, were passed at this time. She suffered, however, almost constantly from pain, jaundice, and impaired nutrition, and finally died on the first of January last.

At the autopsy, the common biliary duct was found almost absolutely closed. The gall bladder was indurated, atrophied and firmly contracted over two very large concretions.

*Disease of the Kidneys, complicating tuberculous disease of Lungs.*—Dr. Ross exhibited the lungs, trachea and kidneys of a patient, who had died at the county hospital. The principal symptoms were cough, emaciation, œdema of extremities and face, delirium, albuminuria, and vomiting.

The immediate cause of death seemed to be the complete exhaustion produced by the ceaseless vomiting.

At the autopsy, there were found extensive deposit of tubercles, with cavities in the lungs; the kidneys were very large and fatty; the trachea and ileum deeply ulcerated.

*Abscess between the uterus and rectum.*—Dr. Danforth presented the lungs, uterus and ovaries of an unmarried patient, twenty-two years of age, who had some months previously suffered from symptoms of peritonitis, and two weeks before death, from an acute attack of inflammation of the lungs, with obscure abdominal disease.

At the autopsy, the lungs were found studded with fine miliary tubercles, rendering the whole tissue like that found in hepatization. There were extensive tuberculous deposits in the peritoneum. The traces of the former peritonitis were observed in the firm adhesions between the ovaries, uterus and bowels. There was a large sack, partially filled with pus, between the uterus and rectum, and communicating with the latter through quite a large opening.

*Death from Chloroform.*—Dr. Powell presented an account of a fatal case from the use of chloroform. The patient, a young man twenty-two years of age, was apparently in robust health, although he had suffered for a long period from caries of the tibia. An operation for the removal of a sequestrum was deemed necessary, and performed on the morning of the 15th of November last. Although the patient had been directed to take no breakfast, he drank a cup of coffee and ate a cracker.

The chloroform, manufactured by Messrs. Powers & Weightman, was administered from a folded napkin, by a careful assistant, its effects being closely watched by Dr. P., until the commencement of the operation, when it was discontinued. No stimulant was given previous to the anæsthetic. No unfavorable symptom appeared either before or during the operation, which lasted about fifteen minutes. At the close of the operation the patient made an effort to vomit, and on being turned upon the side ceased to breathe. The tongue was at once drawn from the mouth, water dashed in the face, and artificial respiration (M. Hall's method) secured for a few moments, when breathing re-commenced, continued about four minutes and again ceased. Renewed efforts caused the patient to breathe three times, but he again ceased to breathe, no efforts being sufficient to resuscitate him.

An autopsy, eight hours after death, revealed no satisfactory cause of the fatal result. There were a very few minute pieces of cracker in the right bronchial tube. The right side of the heart was greatly distended with blood; the right auricle was thickened; the left side of the heart contracted. The blood in the heart and large vessels was not coagulated. The other organs were in a normal condition.

*Extensive Calcareous Deposit in the Pericardium.*—Doctor Ross presented the heart and pericardium of a male patient, sixty-five years of age, who had died at the County Hospital. The symptoms during the last few weeks of life, were almost constant delirium, irregular respiration, at times almost ceasing, tumultuous action of the heart, although the pulse at the wrist was very feeble, general anasarca, and prostration. The patient died comatose.

More than two-thirds of the whole tissue of the pericardium were so filled with firm calcareous deposits, that the two halves of the membrane after the removal of the heart, retained their form like eggshells. The arch and valves of the aorta were also calcareous. The smaller arteries of the abdomen, limbs, base of the brain, and orbit, were converted to hard, inelastic tubes. The lungs were normal; kidneys atrophied, cysts being present in their substance.

*Cavity in Lung communicating with the Pleural Space.*—Dr. Ross also exhibited the lungs of a male patient twenty-seven years of age, who had died at the county hospital, after having suffered from symptoms of emphysema of lungs, and bronchitis complicated with albuminuria. A short time before death, the patient had suffered from great pain in left side. There was extensive anasarca.

The lungs were found to be extensively emphysematous and œdematous with tubercular deposits in both apices. In the left lung there was quite a large cavity communicating through a minute opening with the pleural space. Through this opening large quantities of purulent matter had escaped between the pleura, causing extensive pleurisy. The kidneys were very large and fatty.

*Medullary Cancer of the Œsophagus.*—The patient, a German physician about fifty years of age, under the care of Dr. Powell, experienced slight difficulty in swallowing. At the end of six weeks, he sought the advice of Dr. Powell, who found the œsophagus so closed that the patient was unable to swallow. For four weeks subsequently the patient was supported wholly by nutritive enemata, dying at the end of this period from exhaustion. The progress of the disease was more than usually rapid. The portion affected was about four inches in length near the middle of the œsophagus, its tissue throughout its whole diameter being implicated, and the passage absolutely closed. No other organs were affected.

*Polypus of the Lachrymal Sack.*—Dr. Holmes reported a case of polypus of the lachrymal sack which he had removed with the assistance of Dr. Weeks. The patient, a female thirty-four years of age, had suffered two years from obstruction of the nasal duct, with the usual symptoms. Pressure upon the tumified sack produced a discharge of mucus and watery fluid through the upper punctum, without, however, causing the disappearance of the tumor. The exact diagnosis was doubtful. The tumor appeared too hard for a polypus, and not sufficiently well defined for a calcareous concretion.

After slitting the lower canaliculus, there was much more difficulty than usual in introducing the probes. Even after the stricture, which was situated near the middle of the duct, had been well dilated, there was, at times, great difficulty in introducing the probe.

There soon appeared a button of polypoid growth through the dilated canaliculus. The patient was advised to have the sack laid open and the tumor removed.

A couple of days after this, while in the act of blowing the nose, the patient suddenly perceived an inflation of the sack, followed by the appearance of a small tumor in the inner angle of the eye. This proved to be a polypus about a sixth of an inch in diameter, spherical in shape, and attached within the sack by a narrow peduncle. The removal of the polypus was effected without difficulty.

*Serious Result of Forcible Dilatation of the Sphincter ani.*—Prof. Davis stated that he had recently been requested to examine a young lady, who had suffered, as she stated, from perineal abscess, and subsequently, fistula in ano, which a surgeon had attempted to relieve by forcible dilatation of the anus. This operation consisted in introducing one or two fingers of each hand into the rectum and stretching the sphincter till its muscular fibres were completely ruptured. In this case, six months after the operation, the sphincter had remained absolutely paralyzed. The unfortunate patient was totally unable to retain the fæces.

Several members stated that they had witnessed the same operation performed for the relief of fissure of the anus, with relief of the disease without unpleasant consequences.

Dr. Davis also reported the case of a healthy laborer—in which he had injected a few drops of strong solution of persulphate of iron in six or eight large varicose tumors, situated between the foot and knee. Hard coagula were speedily produced, one of which near the foot ulcerated and sloughed. In two weeks the patient was able to commence work, which he followed for two months without difficulty, although the coagula were not wholly absorbed. At the end of this period the limb became enormously swollen, very dangerous constitutional symptoms, as in erysipelas, supervening.

Under the use of the muriated tincture of iron and sulphite of soda, the patient recovered perfect use of the leg, although he was in a very dangerous condition several days, and large vesications and abscesses formed, from which were discharged large quantities of pus and *debris* of cellular tissue.

